# Topics of the Month

**Published:** 1896-08

**Authors:** 


### TOPICS OF THE MONTH.

THE Buffalo Fresh Air Mission Hospital, at Athol Springs, was opened July 1, 1896, and will remain open until September for the reception and treatment of young children suffering from cholera infantum and other acute diarrheal diseases. The hospital is on the shore of Lake Erie, ten miles from Buffalo, and is reached by frequent trains on either the Lake Shore or W. N. Y. & P. R. R. Anyone who wishes to send a patient should telephone either to one of the staff physicians or to the Fitch Institute. During the month of July the hospital was in charge of Dr. DeWitt H. Sherman, and during August it will be under the charge of Dr. Irving M. Snow, who will make daily visits from Buffalo. The resident physicians at the hospital are Dr. Nairn and Dr. Mesmer, and a corps of trained nurses is under the superintendence of Miss King, who has been at the hospital for the past two summers. All physicians are familiar with the beneficial effects of absolutely fresh air and correct diet for the diseases treated at this hospital. The physicians of Buffalo are urged to interest themselves in securing the benefits of this charity for those who need them. No charge is made for either mothers or children, but the mothers will be expected to aid to some extent in the care of the building. We commend this deserving charity to the attention of the citizens of Buffalo, whom we hope will respond with befitting liberality to its appeals for aid.

---

THE substitution of the preparation of one manufacturing chemist for that of another by careless or unscrupulous druggists, is an evil that ought to be severely dealt with when the offense is detected, and it is one in which every physician is interested in correcting. When a prescription druggist violates the first ethical principles, either by substituting or counter-prescribing, he is unworthy of the confidence of the medical profession or of the people who patronise him.

The *Pharmaceutical Era* has lately discussed this question in a most dispassionate manner in connection with a complaint made by Fairchild Bros. & Foster, of New York, that their essence of pepsin has been substituted for the preparations of other manufacturers so frequently that forbearance now has ceased to be a virtue. They present the evidence in a manner too strong to admit of doubt. The *Era*, in an editorial comment on the subject, says :

Fairchild Bros. & Foster, by their action, place the charge where it belongs, and this cannot fail to benefit honest dealers. Every honest druggist owes it to himself and his profession to speak plainly on this subject. He should adopt the most strict rules for his own establishment; improve every opportunity to condemn the practice of substituting and see that resolutions to this effect are passed by his local, state and national associations. Each druggist should make it a point to give his physicians and his customers to understand that when a prescription comes into his establishment, it is filled with exactly what it calls for. There can be no middle ground, no compromise, no question on this point.

The importance of this subject to every physician, patient and prescription druggist, cannot easily be overestimated; and all these three groups of parties in interest must unite to prevent such practices in future, and to punish the offender whenever and wherever detected and against whomsoever it may be made.

---

The so-called Christian science craze, a fad that every intelligent man and woman in civilised communities discountenances and only the weak, silly or infamous give support, has received proper chastisement at the hands of the coroner's jury in the Dominion of Canada. The following newspaper dispatch from the *Buffalo Morning Express* of June 30, 1896, tells the story:

Adelaide Maria Goodson, while under the treatment of Mrs. Beer, a Christian scientist, died on June 15th and was buried. Subsequently the remains were exhumed and an inquest held, at which it was shown that the girl had died of diphtheria and had not received any medical treatment. Mrs. Beer prayed silently "against the belief in disease," taking a dollar a prayer, and the patient died. The following verdict was rendered tonight: "That Mrs. Mercy Helen Beer, David Goodson and Mrs. Maria Goodson, on the 15th day of June, 1896, in the city of Toronto, county of York, did feloniously and unlawfully neglect, and thereby kill and slay Adelaide Maria Goodson, against the peace of our lady, the Queen, her Crown and dignity."

We could wish that every coroner's inquest would yield as tangible results and couch its verdicts in as clear and unmistakable English.

---

Dr. James F. W. Ross, of Toronto, suggests a new method in dealing with rupture of the puerperal uterus. He bases this suggestion on a case of traumatic rupture of the uterus at the fourth month lately seen by him in consultation and on which he operated. It appears that through some manipulation to deliver the secundines a rent was made in the uterus which was undiscovered at the time. Some days later when the case was seen by Dr. Ross he found the condition of the patient extreme and advised immediate operation. He opened the abdomen and found the uterus with a rent through its posterior wall reaching to the fundus. The tissues were too friable to suture, so he cleaned out the cavity and made through-and-through drainage with a rope of iodoform gauze. A drainage-tube was introduced into the abdominal wound through which the gauze was drawn down through the uterine rent into the vagina.

The woman made an excellent recovery, and Dr. Ross thinks this method of dealing with ruptured uterus is adequate in cases unsuitable for suture. He is not aware that anyone has before called the attention of the profession to the method here proposed and successfully employed in the case referred to.

---

There is no stronger proof of the value of an efficient health department than the returns Buffalo has received for the money it has invested in establishing hers on a sound basis. Hear the *Buffalo Evening News* on the subject:

BUFFALO'S SANITARY TRIUMPH.

The *New York Times*, under the above head, calls attention to the death-rate of this city for the first six months of this year, which was reported at only 11.67 per 1,000, and says: We could scarcely believe that the figures had been transmitted accurately, but an examination of the news columns of our Buffalo contemporaries proves that there was no error.

Buffalo has astonished the state with the result of judiciously applied sanitary efforts. These efforts, feeble at first, gained strength as the methods introduced showed results. Prejudice against new and unfamiliar ways was met with. Professional jealousies had to be overcome. Old systems had to be rooted out. Scientific applications, puzzling to the nonprofessional lookers-on, are never popular until their efficacy has been shown in well-defined results.

So thorough has the work been performed that Bufialo's sanitary triumph is now attracting the attention of the press of the state and creating profound astonishment among scientific men in the larger cities of the country. The *New York Times*, in the course of its remarks, dwells upon one or two features of the system upon which the health department depends, as follows :

We do not wish to give undue prominence to the precautions relating to the milk supply, but they should not be overlooked. Buffalo is one of the few cities in the world which have undertaken to prevent the sale of milk from "consumptive" cows to their inhabitants. It requires the dairyman, whether his herd be kept in or out of the city, to produce a certificate showing that his cows are not tuberculous. We infer, of course, that this involves the use of the tuberculin test. Other infectious diseases, as well as tuberculosis, are spread abroad by the agency of the milk supply when the milkman's water supply or premises are infected. It will be observed that in Buffalo the premises of all milk dealers are inspected, with reference to this danger to which consumers are exposed, and that the record of cases of illness along the delivery routes must give timely warning as to local epidemics of typhoid or scarlet fever caused by the sale of milk infected with the germs of these diseases.

These safeguards, which should be used in every city, have undoubtedly affected the death-rate in Buffalo. We do not desire to overestimate the importance of them as compared with other sanitary precautions, but it may be pointed out that such care with respect to the milk supply and water supply—exercised as it is in only a few cities—indicates clearly an exceptionally enlightened and effective administration in all branches of municipal and sanitary work. We may be sure that a health department which displays so plainly its progressive spirit has neglected none of the other sanitary precautions with which the people of a majority of civilised cities are familiar.

The people and the government of Buffalo may justly be proud of this very remarkable death-rate, which we think is not equaled in any other city of the world. It is apparent that much credit for this extraordinary showing is due to the municipal health department, but the most enlightened and energetic sanitary officers must have the cordial support of the people if their efforts are to be successful. Such support must have been given in Buffalo to Health Commissioner Wende.

